# A normativity mapping review on end-of-life care in long-term care institutions by authors from Germany, Austria, and Switzerland

**DOI:** 10.1007/s11019-025-10278-3

**Published:** 2025-06-06

**Authors:** Ingrid Metzler, Hanna Mayer, Giovanni Rubeis, Jasmin Eppel-Meichlinger

**Affiliations:** 1 Department of General Health Studies, Division of Biomedical and Public Health Ethics, Karl Landsteiner University of Health Sciences, Dr. Karl-Dorrek-Straße 30, 3500 Krems, Austria; 2 Department of General Health Studies, Division of Nursing Science with focus on Person-Centred Care Research, Karl Landsteiner University of Health Sciences, Dr. Karl-Dorrek-Straße 30, 3500 Krems, Austria; 3 Institute of Ethics and History of Medicine, Greifswald Medical School, Ellerholsstr. 1-2, 17487 Greifswald, Germany

**Keywords:** End-of-life care, Palliative care, Advance care planning, Normativity, Theories of the social, Mapping review

## Abstract

This article presents the findings of a “normativity mapping review” designed to make visible the breadth of normative understandings at work within interdisciplinary scholarship on end-of-life care in long-term care institutions. The scope of the literature was limited to peer-reviewed articles authored by scholars affiliated with institutions in Austria, Germany, and Switzerland. Terms and slogans associated with the hospice movement were used as keywords to search for literature in both German and English. During data analysis, values, frameworks, and actions were systematically extracted from the articles. Actions were then clustered into four groups: one involving actions aimed at planning future end-of-life decision-making; another focused on transforming cultures of care; a third encompassing end-of-life practices; and a fourth consisting of residual actions. A comparison of two of these groups shows that normative understandings of end-of-life care in long-term care institutions take shape around two poles—a procedural pole, involving standardised tools that can be used in specific practices to address challenges and improve care, and a substantive pole, centred on the embodied competencies and moral sensibilities of caregivers in realising visions of good end-of-life care.

## Introduction

Interdisciplinary collaborations have become a cornerstone of contemporary health research. They are perceived as helping to make knowledge more robust (Nowotny et al. 2003). However, such collaborations can also be difficult in practice. Among others, they can be hindered by the differing assumptions that scholars from various fields bring to such conversations. These include assumptions about the nature of human health and illness, the methods required to study them, or the objectives of health research. Within a single discipline, such assumptions often function as tacit forms of normativity, helping scholars collaborate by building on a common ground that can be taken for granted (Cribb 2020). However, when scholars collaborate across disciplinary boundaries, these implicit assumptions can become obstacles to meaningful dialogues, contributing to misunderstanding or conversations at cross purposes (Doornebosch et al. 2022).

In this article, we discuss and use a normativity mapping review, i.e., a method that we have developed to map the aforementioned assumptions—the explicit and implicit forms of normativity at work in scholarly literature in interdisciplinary fields of health research. With normativity we refer to values, frameworks, and understandings of goods and bads that guide social practices (Taylor 2003)—including the epistemic practices of scholars (Haraway 1988; Jasanoff 2004). With explicit normativity, we refer to commitments that are articulated in discourses, e.g., in the forms of values that ethicists use to justify the desirability of practices. With implicit normativity we refer to those more tacit forms of normativity that shape social practices (Taylor 2003). Following Carter (2018, p. 190) we take implicit normativity to consist of “unstated or taken-for-granted assumptions about what is good and bad, right or wrong, required or not required”.

A “normativity mapping review” reviews scholarly literature, systematically extracts values, frameworks, and actions as units of “normative information” (Mertz et al. 2016, p. 7), and compares them to map normativity at work in a field. While values and frameworks help to identify more explicit forms of normativity, actions help to elucidate more implicit forms. In reviews within health research, actions are often understood as interventions and assessed in terms of their measurable outcomes (e.g., Kampstra et al. 2018). In this review, we approach actions not in terms of the goods they demonstrable produce, but in terms of the goods they are expected to produce. We take actions to imply “that things could and should be better than they are” (Carter 2018, p. 189). We approach them as “keyholes” (Hochschild 2016) through which the contours of better worlds—those scholars seek to help realize—can be seen. By extracting, mapping and comparing values, frameworks, and actions, a normativity mapping review aims to make visible the normativity that structures interdisciplinary fields of knowledge. Making such structures visible can help us engage with normative assumptions shaping epistemic practices, make implicit normativity more explicit, and foster interdisciplinary dialogues and collaborations.

We discuss this methodology by engaging with scholarship on end-of-life (EOL) care in long-term care (LTC) institutions authored by scholars from Austria, Switzerland, and Germany—the field that has inspired us to develop a methodology capable of making more tacit forms of normativity visible. EOL care refers to the care provided to individuals in the final phase of life. EOL care in LTC institutions brings together scholars from a range of disciplines, including nursing, geriatrics, medicine, and bioethics.

How residents live and die in LTC institutions is an important issue that benefits from contributions across various disciplines and fields of inquiry. However, scholarship on EOL care exemplifies the challenge of interdisciplinary collaborations. Scholars agree *that* the quality of EOL care in LTC institutions should be improved (Froggatt et al. 2020, Harasym et al. 2021), but they disagree on *how* such improvements could and should be achieved, suggesting a diversity of actions (Van Den Noortgate and Van den Block 2022). The diversity of suggested actions reflects the complexity of the problem. EOL care raises moral dilemmas, such as the permissibility of practices of assisted dying, as well as practical challenges, such as how to care for residents who may no longer be able to express their wishes (Alanazi et al. 2024). These problems become even more pressing in the context of LTC institutions, which are often shaped by resources constraints (Harasym et al. 2020), including shortages of caregivers and time that restricts care practices. However, in this review we argue that the diversity of actions stems not only from the complexity of the problem, but also from varying conceptions of what goods, rights, and better worlds scholars consider worth pursuing.

In this article, we seek to map these normative conceptions with the help of a normativity mapping review. Such a review can enhance our understanding of the sometimes tacit forms of normativity that shape research on EOL care in LTC institutions. We propose that making these underlying normative understanding visible can also improve practice. Making tacit forms of normativity explicit can facilitate dialogues and collaborations between different groups of caregivers involved in EOL care in LTC settings.

## Methods

### Design

In light of our interest in normative dimensions of knowledge production, we built on the major tenets of a “focused mapping review and synthesis” (Bradbury-Jones et al. 2019). Such a review has four primary features. It: (1) focuses on a specific area of knowledge production (rather than the entirety of evidence), (2) explores this in an epistemological context, (3) results in a descriptive map of the characteristics of research (findings), and (4) comments on knowledge production (rather than the state of findings) (Bradbury-Jones et al. 2019).

While we approached EOL care and normativity in a broad way, we restricted the geographic scope of our review. Our goal was to produce a sample of literature that enabled mapping various normative elements within comparable settings. The provision of institutionalized LTC varies significantly between countries, primarily in its organization (by public, for-profit or non-governmental providers), financing (cash benefits, in kind benefits or out-of-pocket payments) and resource generation (via general taxation, mandatory social security and voluntary private insurance) (Flawinne et al. 2023). Within this diverse landscape, Germany, Austria, and Switzerland are comparatively similar, as they are comparably wealthy, have similar care systems, and also share the German language. Moreover, health disciplines in Germany, Austria, and Switzerland often share bodies of knowledge, i.e., each other’s publications, guidelines, and recommendations.

### Eligibility criteria

We were interested in original research articles in English or German in which the authors made normative statements about EOL care in institutionalized LTC. Considering our interest in original research, we excluded literature reviews. We included literature with at least one affiliation with an institution located in Austria, Germany, and/or Switzerland among the authors. We made no restrictions regarding the publication date and included publications from international peer-reviewed journals.

### Search strategy

We used terms and slogans from the “hospice movement”, a social movement that has problematized how societies deal with death and dying (Heller et al. 2012), as search terms. We assumed that the use of terms and slogans from the hospice movement in scholarly literature signalled invitations to improve EOL care and could therefore be used as “markers” for normativity. We conducted a non-systematic, free web search on terms and slogans used by the hospice movement in Austria, Germany, and Switzerland, searching for websites, white papers of associations, societies, and regional sponsors, as well as textbooks that reviewed the historical developments of hospice culture in this regional area (Heller et al. 2012; Spörk and Heller 2012).

For the systematic search, we used MEDLINE, Scopus, and LIVIVO in January 2023 using the following terms and phrases in English and German: “Life to the end”, “Until the end”, “Giving life to the days”, “Human Dying”, “Self-determination”, “Autonomy”, “Quality of Life”, “Dignity”, and “Accompaniment”. We used the setting of institutionalized LTC and the context of the end of life or dying as additional search components. We used wildcards to include different spellings and word endings of the search terms.

### Selection of articles

The articles could be automatically narrowed down to publications with at least one contribution from an author affiliated with an institution in the German-speaking area. Publications were then automatically limited to English or German language and to “article” publication type.

**Fig. 1:**
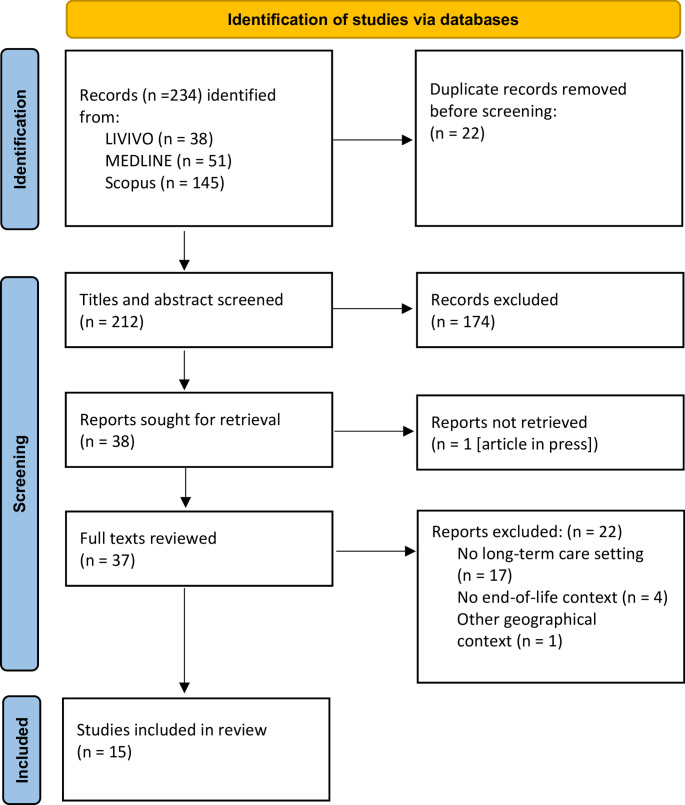
Identification, selection, and inclusion process

Two authors (JEM, IM) independently screened titles, abstracts, and full texts. They discussed discrepancies in the selection process until a consensus was reached (Fig. 1). We used the Systematic Reviews Web App Rayyan QCRI 25 for data management during the selection. Table 1 lists all articles that met our inclusion criteria.

### Data analysis

Our data analysis followed qualitative content analysis strategies (Schreier 2012). We grounded our objective to map normativity in three units of “normative information” (Mertz et al. 2016, p. 7) that we systematically extracted from all articles: (1) values, (2) frameworks, and (3) actions. These units served as primary categories in our data analysis.

The three analytical categories evolved throughout the research process, in which we combined deductive with inductive strategies. While we used values from the beginning, we identified frameworks as a recurring unit of normative information during analysis and subsequently incorporated this category. Initially, we had also planned to use categories like the authors’ imaginations on good EOL care or problematizations. However, as these proved suitable for only a portion of our sample, we discontinued their use. Conversely, we observed that all articles contained actions, likely reflecting the practice-oriented nature of health sciences research. Using actions helped us to accommodate the heterogeneous nature of literature included in our sample, while also allowing us to ground our interest in more implicit forms of normativity in data segments that we could extract from all articles.^1^

Considering our objective to map the scope of normative understandings at work in the scholarly literature, we did not assess the robustness of claims, e.g., by differentiating between empirically grounded or primarily conceptual research. We treated all actions as keyholes to normativity underpinning scholarship. We analysed the articles’ texts. We did not analyse tables, figures, or primary quotes because information was reduced and abstracted to such an extent that the underlying implicit assumptions could not be reconstructed. Subsequently, we merged codes and clustered codes into subgroups. Two authors (JEM and IM) performed the data analysis and synthesis.

## Results

### Characteristics of included articles

We included 15 articles after full text screening (Table 1). Their publication year ranged from 2003 to 2022, with most in 2020 (*n* = 3) and 2021 (*n* = 3). 7 articles were published in interdisciplinary journals. 4 articles were published in nursing journals; 3 in medical journals.^2^ Authors affiliation was associated with departments of palliative care (*n* = 11), social sciences (*n* = 10) and nursing (*n* = 9) (Table 2).

**Table 1:** Included articles

Authors, year	Country	Aim of the study
Scorna et al. 2022	Germany	To examine, from an ethical perspective, the processes that take place when people are dying and are transferred
Frewer-Graumann and Teichmüller, 2021	Germany	To investigate the cooperation between an elderly care facility and an outpatient hospice service
Stängle et al. 2021	Germany, Switzerland	To assess the experiences, personal attitudes, and professional stances of Swiss health care professionals toward voluntary stopping of eating and drinking
Droz Mendelzweig 2020	Switzerland	To investigate how advanced directive interventions in nursing homes strive simultaneously to behave in line with the principles of care ethics
Klemmt et al. 2020	Germany	To explore wishes and needs, such as existing and preferred communication processes, of residents and relatives regarding medical and nursing planning at the end of life
Groebe et al. 2019	Germany	To get insights into specific requirements and conditions for communication about the end of life in various EOL care settings
Klemmt et al. 2021	Germany	To provide information about the content of living wills of nursing home residents
van Oorschot et al. 2018	Germany	To systematically determine the kind of care and support that nursing home residents desire
Stängle et al. 2020	Germany, Switzerland	To assess the incidence of voluntary stopping of eating and drinking in long-term care and to gain insights into the attitudes of long‐term care nurses
Reitinger et al. 2018	Austria	To highlight some of the most important insights into themes and issues that relatives of persons having died in a nursing home find essential
Bollig et al. 2017	Austria, Germany	To explore ethical challenges and approaches to implement systematic ethics work in nursing homes
George 2015	Germany	To describe the nursing, medical and psychosocial situation of dying people in nursing homes and formulate suggestions for improvement
Lederer et al. 2015	Austria	To implement a palliative treatment plan in nursing homes, and to test its efficiency
Pleschberger 2007	Austria	To explore the meaning of dignity with regard to EOL issues from the perspective of older nursing home residents in western Germany
Heller et al. 2003	Austria	To investigate under which conditions quality of life of dying persons in long-term care can be maintained until the end and the care system can be aligned with the guiding category of dignity

**Table 2:** Publication characteristics of included studies

Total	*n* (%)
	15 (100%)
Year	
2003	1
2007	1
2015	2
2017	1
2018	1
2019	2
2020	3
2021	3
2022	1
Affiliated country*	
Germany	30
Austria	15
Switzerland	8
Journal	
Zeitschrift für Gerontologie und Geriatrie	3
Age and Ageing	1
American Journal of Hospice & Palliative Medicine	1
BMC Palliative Care	1
Deutsche Medizinische Wochenschrift	1
Journal of Advanced Nursing	1
Journal of Clinical Nursing	1
Journal of Pain and Symptom Management	1
Journal of Research in Nursing	1
MMW - Fortschritte der Medizin	1
Pflege und Gesellschaft	1
Scandinavian Journal of Caring Sciences	1
Wiener Medizinische Wochenschrift	1
Affiliated department*	
Palliative care and organisational ethics	11
Social sciences	10
Nursing	9
Palliative medicine	6
Health care research	4
Oncology	2
Gerontology	2
Anaesthesiology and intensive care	2
Economy and management	1
General medicine	1
Education	1
Applied ethics	1
No data	5

*Multiple countings

### Values

The articles used a broad range of values (Table 3), most often without defining them (but see Heller et al. 2003; Pleschberger 2007).

**Table 3:** Values that articles mentioned at least once

	Scorna et al. (2022)	Frewer-Graumann et al., 2021	Stängle et al. 2021	Droz Mendelzweig 2020	Klemmt et al. 2020	Groebe et al. 2019	Klemmt et al. 2021	van Oorschot et al. 2018	Stängle et al. 2020	Reitinger et al. 2018	Bollig et al. 2017	George 2015	Lederer et al. 2015	Pleschberger 2007	Heller et al. 2003	Total	% of articles mentioning value
Autonomy and self-determination	x	x	x	x	x	x	x		x		x		x	x	x	12	80,00
Dignity	x		x		x	x	x		x	x	x	x		x	x	11	73,33
Respect	x		x	x		x			x	x	x		x	x	x	10	66,67
Inclusion/involvement/ participation	x	x			x	x				x	x	x			x	8	53,33
Quality of care	x					x				x	x	x	x	x	x	8	53,33
Trust	x			x	x	x		x							x	6	40,00
Well-being	x				x	x					x	x			x	6	40,00
Quality of life	x	x				x						x			x	5	33,33
Collaborations	x	x									x	x				4	26,67
Justice	x			x							x		x			4	26,67
Beneficence	x										x		x			3	20,00
Empathy	x				x					x						3	20,00
Preparedness					x						x		x			3	20,00
Recognition				x								x		x		3	20,00
Compassion	x									x						2	13,33
Freedom				x	x											2	13,33
Holism		x				x										2	13,33
Peacefulness			x						x							2	13,33
Privacy	x										x					2	13,33
Diversity										x						1	6,67
Health					x											1	6,67
Loyalty													x			1	6,67
Quality of death	x															1	6,67
Solidarity										x						1	6,67
Transparency												x				1	6,67

The most frequently used values were autonomy and self-determination, which were used to sustain a broad range of actions. Among others, autonomy sustained using advance directives and justified voluntary stopping of eating and drinking (VSED) as an EOL practice (Stängle et al. 2020, 2021). Several articles referred to autonomy as an endangered value (Heller et al. 2003; Bollig et al. 2017; Klemmt et al. 2020), which was threatened due to impaired cognition or structures, such as time constraints in LTC institutions.

The second most frequently used value was dignity. Dignity was very often used as an adjective, such as in “dignified death” (Pleschberger 2007) or “dignified dying” (Stängle et al. 2020, 2021). Similar to autonomy, dignity was described as a value endangered by structural forces, such as the lack of resources in LTC institutions (Bollig et al. 2017) or ageism (Pleschberger 2007).

### Frameworks

The articles also used *sets of values*,* principles and practices*, as well as approaches, which we categorized as frameworks. These frameworks were more frequently defined than single values. Articles linked them to other scholars’ research, the guidelines of professional societies, or regulations. Several articles used more than one framework (see Table 4).

First, 53,3% of the articles referred to the framework of advance care planning (ACP) (Bollig et al. 2017; van Oorschot et al. 2018; Groebe et al. 2019; Droz Mendelzweig 2020; Klemmt et al. 2020; Klemmt et al. 2021; Scorna et al. 2022; Lederer et al. 2015).^3^ ACP was described as: a “process of planning for future medical care (…), particularly for situations when the patient becomes unable to participate in decisions about treatment and care” (Lederer et al. 2015, p. 988); or, following Rietjens and colleagues (2017) as “the ability to enable individuals to define goals and preferences for future medical treatment and care, to discuss these goals and preferences with family and healthcare providers, and to record and review these preferences if appropriate” (Klemmt et al. 2020, p. 2664).

**Table 4:** Frameworks used in articles

		Scorna et al. (2022)	Frewer-Graumann et al., 2021	Stängle et al. 2021	Droz Mendelzweig 2020	Klemmt et al. 2020	Groebe et al. 2019	Klemmt et al. 2021	van Oorschot et al. 2018	Stängle et al. 2020	Reitinger et al. 2018	Bollig et al. 2017	George 2015	Lederer et al. 2015	Pleschberger 2007	Heller et al. 2003	Total	% of articles men
**(1) Advance care planning**		**x**			**x**	**x**	**x**	**x**	**x**			**x**		**x**			**8**	**53**,**3**
**(2) Variations of palliative or hospice care**		**x**	**x**		**x**		**x**		**x**		**x**	**x**	**x**	**x**	**x**	**x**	**11**	**73**,**3**
Palliative (health) care			x		x		x				x	x		x	x	x	8	53,3
* Palliative Versorgung*		x							x				x				3	20,0
Palliative medicine		x							x								2	13,3
Palliative and hospice care, cultures/philosophy			x				x				x	x			x	x	6	40,0
(3)** Care approaches**		**x**	**x**			**x**	**x**				**x**	**x**	**x**		**x**	**x**	**9**	**60**,**0**
Need-based		x	x			x					x		x			x	6	40,0
Patient-centred		x					x				x	x					4	26,6
Person-centred							x				x						2	13,3
Subject-, target group or individualized			x			x	x										3	20,0
Dignity oriented															x		1	6,67
Caring institutions											x						1	6,67
EOL care in the community											x						1	6,67
**(4) Ethics approaches**		**x**			**x**	**x**					**x**	**x**					**5**	**33**,**3**
Principles of biomedical ethics		x				x						x					3	20,0
Care ethics					x						x	x					3	20,0
EOL care ethics					x												1	6,67
Palliative care ethics												x					1	6,67
**(5) Right to die framework**										**x**							**1**	**6**,**67**

A second set of frameworks involved different combinations of the adjectives “palliative” and “hospice” (*hospizlich*), used by 73,33% of the articles (See Table 4). The meaning of this second set of frameworks was significantly more fluid than ACP, ranging from the descriptive naming of a field of care to prescriptions on how to take care. For instance, “palliative care” and “palliative medicine” were often used without definitions or references and denoted a field of care (Frewer-Graumann and Teichmüller 2021). By contrast, the combination of palliative with “hospice” or with terms such as “culture” or “philosophy” combined descriptions of a field of care with prescriptions of how to take care. For instance, “hospice accompaniment and palliative care” was described as the “practices of the hospice movement”, which aim “to maintain the quality of life until the end through a holistic view of the dying” (Frewer-Graumann and Teichmüller 2021, p. 13), involving a “radical orient[ation] towards the needs of those affected and their relatives” (Frewer-Graumann and Teichmüller 2021, p. 13). Similarly, the use of “culture” and “philosophy” marked more prescriptive understandings of care. One article explained that a palliative culture would consist in the integration of palliative care into the standard care of LTC institutions (Heller et al. 2003), describing palliative care as an “interdisciplinary, interprofessional and interorganizational care concept”, which is “oriented towards proactive decisions to ensure quality of life and dignity in the sense of a socio-psychosomatic and spiritual understanding until the end” (Heller et al. 2003, p. 361).

A third set of frameworks consisted of care concepts and approaches. Such frameworks were often used to emphasize that good EOL care went beyond addressing residents’ medical needs and preferences. For instance, “holistic and individualized care” relied on “[k]nowing about a broad range of psychosocial, spiritual, and existential aspects along with preferences for physical care” (Groebe et al. 2019, p. 2). Dignity-oriented approaches underlined that “‘people matter as individuals, for who they are and not what they can do’” (Pleschberger 2007, p. 201). While they emphasized, that residents were more than bodies, they differed in how they situated these bodies. They ranged from care approaches that emphasized that care ought to be oriented towards the needs of residents, the “affected persons and their relatives” (Frewer-Graumann and Teichmüller 2021) or the “family system” (George 2015, p. 24) to approaches that conceptualized care on the level of the institution, such as the framework of “caring institutions” (Reitinger et al. 2018)—linked to the work of Tronto (2010)—or the community, such as the concept of “compassionate end-of-life care in the community” (Kellehear 2005; Reitinger et al. 2018). The latter emphasized that the responsibility for EOL ought to be shared in “communities” or societies, rather than being “delegated to nursing homes, hospitals and hospices, [or] pushed back into the private sphere” (Reitinger et al. 2018, p. 249).

Moreover, fourth, a third of all articles also referred to one or several ethics frameworks. These included the principles of biomedical ethics (Bollig et al. 2017; Klemmt et al. 2020; Scorna et al. 2022), “care ethics” (also referred to as “nursing ethics” or “relational ethics”) (Bollig et al. 2017; Reitinger et al. 2018; Droz Mendelzweig 2020), “palliative care ethics” (Bollig et al. 2017), and the “ethics of end-of-life care” (Droz Mendelzweig 2020). The principles of biomedical ethics were described as “respect for autonomy, nonmaleficence, beneficence, and justice” (Bollig et al. 2017, p. 2)–with autonomy being described as especially important. The latter was deemed to be challenged in LTC institutions, e.g., in situations in which residents were no longer able to articulate their wishes. In those cases, “care ethics”, “based on relations and the reflection of nursing practices” and the understanding of human selves as “fundamentally connected beings” (Bollig et al. 2017, p. 11), could help provide better EOL care.

A final fifth frameworks was the one of a “right-to-die society” (Stängle et al. 2020), where a desire to die could be openly communicated.

### Actions

The articles mentioned many possible actions that could improve EOL care in LTC institutions. We ordered them into four groups (see Table 5). Three of them were tied to a particular framework: The first group (planning EOL decision making) involved actions tied to ACP. The second group (transforming cultures of care) concerned transformations in LTC institutions to implement frameworks such as palliative care. The third group (accompanying residents’ EOL practices) extended the scope of EOL practices to assisted dying. A final fourth group comprised residual actions, which were not tied to a particular framework.

#### Planning end-of-life decision making

The first group of actions focused on taking actions in the present to manage EOL decision making in the future (Lederer et al. 2015; Bollig et al. 2017; van Oorschot et al. 2018; Droz Mendelzweig 2020; Klemmt et al. 2020; Klemmt et al. 2021). Actions in this group were often seen as answers to specific problems, such as medically unnecessary and socially disruptive hospitalizations or conflicts between groups of caregivers. They were often tied to the framework of ACP.

These actions included the use of particular documents or “tools” (Droz Mendelzweig 2020), such as living wills (Lederer et al. 2015; van Oorschot et al. 2018; Klemmt et al. 2021) or advance directives (Lederer et al. 2015; Droz Mendelzweig 2020; Klemmt et al. 2020); guidelines, e.g., to reduce the number of hospitalizations (Scorna et al. 2022); or invitations to make concepts for ACP (Bollig et al. 2017; van Oorschot et al. 2018; Klemmt et al. 2021). They were described as serving multiple purposes. Among others, they could avoid hospitalizations (Lederer et al. 2015; van Oorschot et al. 2018), safeguard residents’ autonomy (Lederer et al. 2015; Klemmt et al. 2021), prevent conflicts, reduce stress and anxiety, ensure the satisfaction of patients, families, and caregivers (Lederer et al. 2015), and promote dialogues and cooperations between professional caregivers, residents, and their relatives (Droz Mendelzweig 2020).

**Table 5:** Actions discussed or recommended in articles

		Scorna et al. 2022	Frewer-Graumann et al. 2021	Stängle et al. 2021	Droz Mendelzweig 2020	Klemmt et al. 2020	Groebe et al. 2019	Klemmt et al. 2021	van Oorschot et al. 2018	Stängle et al. 2020	Reitinger et al. 2018	Bollig et al. 2017	George 2015	Lederer et al. 2015	Pleschberger 2007	Heller et al. 2003
**Group 1: Planning end-of-life decision making**		x			x	x		x	x			x		x		
Use of documents, such as living wills or advance directives					x	x		x	x					x		
Use of guidelines		x			x											
Use advance care planning					x	x		x	x			x		x		
**Group 2: Transforming cultures of care**			x								x	x				x
Fostering palliative care culture			x								x	x				x
Ethics consultations																x
Cooperation with hospice care services			x													
Creating structures to involve relatives											x					
**Group 3: End-of-life practices**										x						
Discuss voluntary stopping of eating and drinking (VSED) as end-of-life practice										x						
**Group 4: Residual actions**		x	x	x		x	x		x	x	x	x	x	x	x	
Improve conversations, dialogues and counselling			x			x	x									
Ethics work		x										x				
Train professional caregivers		x				x	x		x	x			x	x		
Involve and include relatives		x				x	x				x	x	x			
Enhance collaboration between groups of professional and lay caregivers		x	x								x		x	x		
Collaboration with external actors		x	x	x						x			x			
Increase resources		x					x		x		x					
Improve guidelines/regulations				x			x		x							
Monitor quality													x			
Perform more research			x			x	x						x		x	

#### Transforming cultures of care

The second group of actions concerned more comprehensive transformations in the cultures of care of LTC institutions to improve EOL care. They focused on implementing a specific framework, such as a “palliative care culture” (Reitinger et al. 2018) or a “hospice philosophy” (Frewer-Graumann and Teichmüller 2021) in LTC institutions.

One article described the “cooperation with an outpatient hospice care service” (Frewer-Graumann and Teichmüller 2021) as a way to establish a palliative care culture in LTC facilities. Such a collaboration could improve how dying is dealt with, help caregivers develop hospice competences, and also support relatives (Frewer-Graumann and Teichmüller 2021). Another article highlighted the involvement of relatives as a means of transforming care cultures and as an indicator of a good care culture (Reitinger et al. 2018), framing relatives “person[s] in need (to be cared for)” and as “co-carer[s]” (Reitinger et al. 2018, p. 244). One article described the use of “ethics consulting as an instrument of anticipatory decision-making” (Heller et al. 2003, p. 363) as an action that could support the implementation of a palliative culture.

#### Accompanying residents’ end-of-life practices

A third group of actions consisted in the invitation to discuss “voluntary stopping of eating and drinking” (VSED) (Stängle et al. 2020) as an EOL practice. VSED was described as “the autonomous decision of a discerning person (…), [who] stops the intake of food and liquid with the intention of prematurely ending [their] life” (Stängle et al. 2020, p. 527). While professionals’ understanding of and attitudes towards VSED were reported to be ambivalent, VSED was conceptualized as an EOL practice that could protect autonomous decision-making of a resident to end their life and enabled dignified dying (Stängle et al. 2020).

#### Residual actions

A final group of residual actions comprised actions that were not aligned with any specific normative framework. They ranged from transformations of practices inside LTC institutions, over collaborations with external actors, to actions that policymakers were invited to take.

Several articles underlined the importance of creating structures and spaces to facilitate conversations and dialogues, thus embracing the practice of talking (also salient in the first and second group) while extending it beyond the tabooed issue of death and dying (Groebe et al. 2019). Two articles also discussed how ethics works, e.g., as “systematic ethics work”—“‘the (.) systematic use of different measures, tools and places to enhance ethics discussions and ways to handle ethically difficult situations and choices in nursing homes’” (Bollig et al. 2017, p. 2)—could improve EOL care (Scorna et al. 2022).

A very prominent type of actions concerned training and qualifying professional caregivers (George 2015; Lederer et al. 2015; Groebe et al. 2019; van Oorschot et al. 2018; Klemmt et al. 2020; Stängle et al. 2020; Scorna et al. 2022).

Several types of actions involved collaborations. These included: involving and including relatives (George 2015; Reitinger et al. 2018; Groebe et al. 2019; Klemmt et al. 2020; Scorna et al. 2022); enhancing collaborations between different groups of (professional and lay) caregivers (George 2015; Lederer et al. 2015; Reitinger et al. 2018; Frewer-Graumann and Teichmüller 2021; Scorna et al. 2022); and encouraging collaborations with external actors (George 2015; Reitinger et al. 2018; Frewer-Graumann and Teichmüller 2021; Scorna et al. 2022)—including: general practitioners (George 2015; Scorna et al. 2022), ACP counsellors or outpatient hospice services (Frewer-Graumann and Teichmüller 2021). Two articles called for fostering networks with “caring communities” (George 2015; Reitinger et al. 2018).

Another set of actions addressed the responsibilities of policymakers, as these involved the structures in which LTC institutions were embedded. Such actions included calls for the provision of appropriate financial and personal resources (van Oorschot et al. 2018; Reitinger et al. 2018; Groebe et al. 2019; Scorna et al. 2022) or the improvement of professional guidelines or legal regulations (van Oorschot et al. 2018; Groebe et al. 2019; Stängle et al. 2021). One article recommended to assess and monitor the quality of care (George 2015).

Several articles also suggested research as an action to improve EOL care (Pleschberger 2007; George 2015; Groebe et al. 2019; Klemmt et al. 2020; Frewer-Graumann and Teichmüller 2021; Klemmt et al. 2021). Research could help understand the value of particular instruments (Groebe et al. 2019; Klemmt et al. 2020, 2021) or learn more on the wishes and needs of residents (and their relatives) (Pleschberger 2007; Frewer-Graumann and Teichmüller 2021; Klemmt et al. 2021).

## Discussion

In this review, we extracted values, frameworks and actions from a sample of the interdisciplinary literature on EOL care in LTC institutions to develop a better understanding of both explicit and implicit forms of normativity at work in this body of scholarship.

Our analysis showed that scholars from various disciplines have contributed to the literature on EOL care in LTC institutions, with no single discipline dominating the study of EOL care in these settings. The relatively small size of our sample (*n* = 15) and the slow increase in the number of articles published per year suggest that EOL care in LTC institutions is a fairly neglected topic in scholarly literature. However, the COVID-19 pandemic, which has affected residents and caregivers in LTC institutions, might also have had an impact on the relative neglect of this topic, as other topics, such as protecting the health of residents, might have become more urgent.

We have extracted values, frameworks, and actions from the sample. Values such as autonomy and self-determination as well as dignity were used by many articles, very often without definition and, notably, to sustain the desirability of often very different actions. This shows that values can be interpreted very differently. We take this to suggest that extracting single values from a body of literature is not the most informative category when mapping normativity. Values gain their meaning from the context in which they are used, e.g., the practice or actions they are deemed to justify. The same value can be used to support very different actions, limiting the utility of values as a standalone category of normativity.

We have also deduced frameworks, i.e., sets of values and practices, that were often linked to references such as scholarly literature or guidelines. Frameworks were more helpful for mapping normativity. While several articles used more than one of them, the framework of ACP and palliative care frameworks were significantly more often used than others. The meaning of palliative care frameworks was comparatively fluid. It ranged from descriptions of a field of care to prescriptions of good care. More prescriptive uses were often tied to uses of terms such as “cultures” or “philosophies”.

A comparison of these frameworks reveals important differences in how they conceptualize residents, their relationships with family members, and their caregivers. The framework of ACP, for example, often approaches residents as future patients whose autonomy in EOL decision-making should be safeguarded. In contrast, frameworks associated with palliative care culture and some care models—such as person-centred care—advocate for a broader view of residents as persons rather than merely patients, emphasizing their relational embeddedness. Moreover, the frameworks differ in the degree to which they locate the responsibility for good care in individual caregivers, LTC institutions, or society. These differences suggest that distinct theories of the social (Fay 1996; Latour 2007)—namely, varying understandings of human selves and their entanglement in relationships and structures—are at work in the literature.

The frameworks also help to situate normativity in scholarly research, as their usage appeared to follow temporal and geographic patterns. Given the small size of our sample, the following interpretation should be approached with caution. Nonetheless, the right-to-die framework was invoked in only one article authored by researchers based in Switzerland, while this framework—and the broader topic of assisted suicide—was absent from the other articles. This likely reflects differing national attitudes toward assisted suicide among the three countries represented in this review. Additionally, we observed that palliative care frameworks spanned a wide range, from descriptive accounts of care practices to prescriptive visions of good EOL care. The latter, often articulated through terms such as “palliative care cultures” or “philosophies”, were predominantly found in earlier studies, particularly those authored by researchers affiliated with Austrian research institutions. In contrast, the framework of ACP has become more prominent in recent literature, likely mirroring its rising importance in policy and regulatory discussions. It also appears to be the preferred framework among authors affiliated with German institutions. These temporal and geographic patterns suggest that the normativity at work in scholarly literature reflects the context in which scholarship emerges.

We have also extracted actions within the literature, treating them as signifiers of more implicit forms of normativity. Many of these actions were shared across the sample. For instance, the view that residents should be encouraged to articulate their preferences, wishes, and needs emerged as a common theme across all groups of actions. Likewise, there was broad agreement that communication and training of caregivers are essential for improving the quality of EOL care. A recurrent tenet throughout the literature is the recognition that improving EOL care is also a political issue and responsibility—linked to the allocation of resources to LTC institutions.

However, there were also notable differences, specifically between the first group of actions, tied to the framework of ACP, and the second group of actions, aligned with the framework of palliative care cultures. A comparison of these two groups can help us reflect on the dimensions of more implicit forms of normativity. The two groups of actions differed in how they conceptualized improvements. Actions in the first group were often suggested to mitigate specific problems, such as conflicts between caregivers or unnecessary hospitalizations. In this group, improving EOL care was about reducing such foreseeable bads through specific practices and procedures. More than being guided by imaginations of good or right EOL care, these actions appeared to be tied to efforts to reduce bads and wrongs. Improving EOL care was a matter of avoiding poor practices and addressing problems. The second group of actions, tied to the framework of palliative care culture, was not so much about addressing specific bads or wrongs, but about implementing imaginations of good and right EOL care, such as palliative care cultures. These contrasting orientations towards improving EOL care suggest that normativity can be grounded either in the management of bads and harms or in the pursuit of normative ideals.

Moreover, the two groups also differed in how they envisioned improvements to occur. Actions associated with ACP involved tools—such as advance directive forms—that caregivers could use in specific practices. In this first group, enhancing EOL care was seen as a matter of introducing tools into LTC institutions. In contrast, the second group made no use of such tools. Instead, its actions focused on developing healthcare professionals’ skills and competences, emphasizing the value of adding caregivers with embodied expertise and particular moral sensibilities to LTC settings. Here, good EOL care was not embedded in tools, but in people—their practical know-how, tacit knowledge, and moral sensibilities. In one group, normativity was embedded in non-human tools designed to improve human practices; in the other, it was inextricably tied to embodied knowledge and moral sensibilities of people.

These differences suggest that the normativity at work in this body of literature takes shape between two poles: a more procedural pole, involving standardized tools that people can use in specific practices to address problems and improve EOL care, and a more substantive pole, which centres on the embodied competences and moral sensibilities of caregivers to realize imaginations of good EOL care. The former frames improvement in terms of adding tools and applying standards to reduce problems, while the latter emphasizes affective engagements within situated contexts.

When reflecting on implications for future research, it is important to acknowledge the potential limitations of our study. First, we relied on terminology rooted in the hospice movement to identify relevant articles across multiple disciplines. As a result, we may have overlooked contributions from scholars who use different terms. Second, we excluded non-peer-reviewed literature from our sample, possibly marginalizing perspectives from researchers who publish outside of peer-reviewed journals. Third, we focused on literature from three countries, based on the assumption that their healthcare systems are comparable and that they share a common body of knowledge. However, our findings revealed geographic differences within the sample, highlighting the situated nature of normativity in scholarly literature. Accordingly, we do not claim that our findings can be generalized beyond the contexts of the three countries included in this study.

Future explorations of the breadth of normativity in EOL care in LTC institutions could benefit from incorporating literature from additional countries. Moreover, a normativity mapping review could be expanded to other materials and data, such as policy documents or position papers. Such an approach could help to both broaden and refine the map of normativity at work in the field of EOL care in LTC settings.

## Conclusions

We developed and used a normativity mapping review to map the normativity at work in the literature on EOL care in LTC institutions from authors with Austrian, German, or Swiss affiliations.

We showed that normativity on EOL care in LTC institutions takes shape in between two poles— a procedural pole focusing on tools that caregivers can use to improve EOL care by addressing problems, and a more substantive pole centred on the embodied skills, competences, and moral attitudes that caregivers need to realize visions of good EOL care.

Moreover, our research also showed that extracting values, frameworks and actions from a body of literature is a valuable methodology for mapping normativity. Using actions as analytical category can enhance engagements with more implicit forms of normativity, particularly in practice-based field such as health sciences.

Such a normativity mapping review can also help improve practices in interdisciplinary fields, such as EOL care in LTC institutions. Collaborations between different fields can be complicated by the differing and often tacit normative assumptions that scholars bring to such conversations. Making these implicit understandings more explicit can facilitate conversations and collaborations. These discussions need not determine which forms are more appropriate, desirable, or justified, as different understandings of normativity are not mutually exclusive. However, making them explicit can help develop common ground on how different approaches might be combined—for instance, through divisions of labour—to improve residents’ living and dying experiences in LTC institutions. This can enhance interdisciplinary collaborations, transforming tacit forms of normativity from obstacles into resources for collective engagement.

## Footnotes

1 The actions differed in form. The first type involved descriptions of experiences with specific actions which often appeared in the results sections of empirical articles. The second type took the form of calls for transformation or recommendations, typically found in the discussion sections of empirical articles or in conceptual articles. A third type involved descriptions of good and desirable practices. For this review, we extracted actions of the first and second types.

2 One journal did not indicate its target audience.

3 ACP was also referred to as “*Vorausplanung zum Lebensende*” (in German) or “advance planning of medical and nursing care” (Klemmt et al. 2021).
